# Habitual salt preference worsens blood pressure in hospitalized hypertensive patients with omicron infection under epidemic-related stress

**DOI:** 10.1186/s12889-023-17633-0

**Published:** 2024-01-09

**Authors:** Chenyi Wang, Wanhong Tan, Xiaoxiao Liu, Miao He, Shi Zeng, Maojie Sun, Lijuan Yan, Min Li, Kun Zhan, Kaifa Wang, Qiang Li

**Affiliations:** 1 Department of Urology Surgery, Daping Hospital, Army Medical University, 400042 Chongqing, China; 2 Center for Hypertension and Metabolic Diseases, Department of Hypertension and Endocrinology, Daping Hospital, Chongqing Institute of Hypertension, Army Medical University, 400042 Chongqing, PR China; 3 Chongqing Yuzhong District Daping Street Community Health Service Center, 400042 Chongqing, PR China; 4 Department of Neurosurgery, People’s Hospital of Chongqing Banan District, 401320 Chongqing, PR China; 5 Department of Pharmacy, The Seventh People’s Hospital of Chongqing, 400054 Chongqing, PR China; 6 School of Mathematics and Statistics, Southwest University, 400715 Chongqing, PR China; 7 Department of Nephrology, Beijing Key Laboratory of Kidney Disease Research, First Medical Center of Chinese PLA General Hospital, 100853 Beijing, China

**Keywords:** Habitual salt preference, Blood pressure, Omicron, Psychosocial stress

## Abstract

**Background:**

We investigated the synergistic effect of stress and habitual salt preference (SP) on blood pressure (BP) in the hospitalized Omicron-infected patients.

**Methods:**

From 15,185 hospitalized Omicron-infected patients who reported having high BP or hypertension, we recruited 662 patients. All patients completed an electronic questionnaire on diet and stress, and were required to complete morning BP monitoring at least three times.

**Results:**

The hypertensive group (*n* = 309) had higher habitual SP (*P* = 0.015) and COVID-19 related stress (*P* < 0.001), and had longer hospital stays (7.4 ± 1.5 days vs. 7.2 ± 0.5 days, *P* = 0.019) compared with controls (*n* = 353). After adjusting for a wide range of covariates including Omicron epidemic-related stress, habitual SP was found to increase both systolic (4.9 [95% confidence interval (CI), 2.3–7.4] mmHg, *P* < 0.001) and diastolic (2.1 [95%CI, 0.6–3.6] mmHg, *P* = 0.006) BP in hypertensive patients, and increase diastolic BP (2.0 [95%CI, 0.2–3.7] mmHg, *P* = 0.026) in the control group. 31 (8.8%) patients without a history of hypertension were discovered to have elevated BP during hospitalization, and stress was shown to be different in those patients (*P* < 0.001). In contrast, habitual SP was more common in hypertensive patients with uncontrolled BP, compared with patients with controlled BP (*P* = 0.002).

**Conclusions:**

Habitual SP and psychosocial stress were associated with higher BP in Omicron-infected patients both with and without hypertension. Nonpharmaceutical intervention including dietary guidance and psychiatric therapy are crucial for BP control during the long COVID-19 period.

## Introduction

Since first being discovered in Southern Africa in November 2021, there has been over 770 million confirmed cases with severe acute respiratory syndrome coronavirus 2 (SARS-CoV-2) globally as of September 2023. The Omicron variant is still the dominant strain globally, which seemed to have evolved toward less virulence [1, 2], with a higher rate of severe outcomes and considerable mortality reported in unvaccinated people, especially older adults [3]. A considerable number of those with severe outcomes had cardio-metabolic diseases, such as hypertension, diabetes, coronary heart disease, and obesity [4–6]. Earlier studies had found that hypertension was the most common underlying disease, and was the most important risk factor for viral clearance, with an up to 2.5-fold higher risk for both severity and mortality in COVID-19 patients [7–9]. Hobbs et al. found that stricter blood pressure (BP) control in patients with hypertension did not appear to reduce the risk of COVID-19 complications [10]. Furthermore, it was reported that the COVID-19 pandemic worsened BP control, even in patients who perceived no marked change in their diet or exercise [11]. Thus, studying the factors influencing BP in patients during the Omicron epidemic is important to accumulating experience in preventing disease progression and improving patient resilience.

A remarkable growth in psychosocial stress was expected to occur during the COVID-19 pandemic [12]. High salt intake is an important risk factor associated with elevated BP and is related to psychological stress. Experimental studies have found that early high salt exposure may increase the risk of stress-related neuropsychiatric disorders and stress sensitivity [13, 14]. In the USA, a significant increase in BP was observed during and after the COVID-19-related lockdown [15], which was explained by the authors as partially due to the emotional stress of the lockdown. It has been suggested that the abrupt lockdown of populations induced stress that would foster addiction-related habits, and emerging evidence has pointed to a major shift to consumption of high-sodium foods during the pandemic lockdown in populations from different countries and cultures [16–18]. Such addictive habits are related to taste signal processing and hedonic responses to foods, and can be measured by salt preference (SP) [19]. China is traditionally a high salt consumption country, with a mean daily salt intake per person of around 11.0 g to 14.0 g [20]. Furthermore, Chinese people are prone to sodium sensitivity-related increased BP, which is prospectively associated with incidence of hypertension [21]. Our previous work found that habitual SP was related to the salt intake and metabolic activity in the central gustatory system [22]. It remains uncertain whether habitual salt preference contributed to the deterioration of blood pressure control in hypertensive patients during the COVID-19 pandemic.

Therefore, we aimed to investigate the potential synergistic effect of stress and habitual SP on BP levels in hospitalized Omicron-infected hypertensive patients during the COVID-19 pandemic. We hypothesized that the stress induced by the Omicron epidemic, coupled with habitual SP, may contribute to elevated BP levels, which could result in a poorer prognosis and potential health hazards for hypertensive patients. To assess habitual SP, hypertensive patients completed a diet questionnaire based on our previous report [22]. Additionally, we used the Hamilton Anxiety and Depression Scale (HAMA, HAMD) questionnaires to evaluate the levels of anxiety and depression among patients in the makeshift hospital.

## Methods

### Study design and participants

**Fig. 1:**
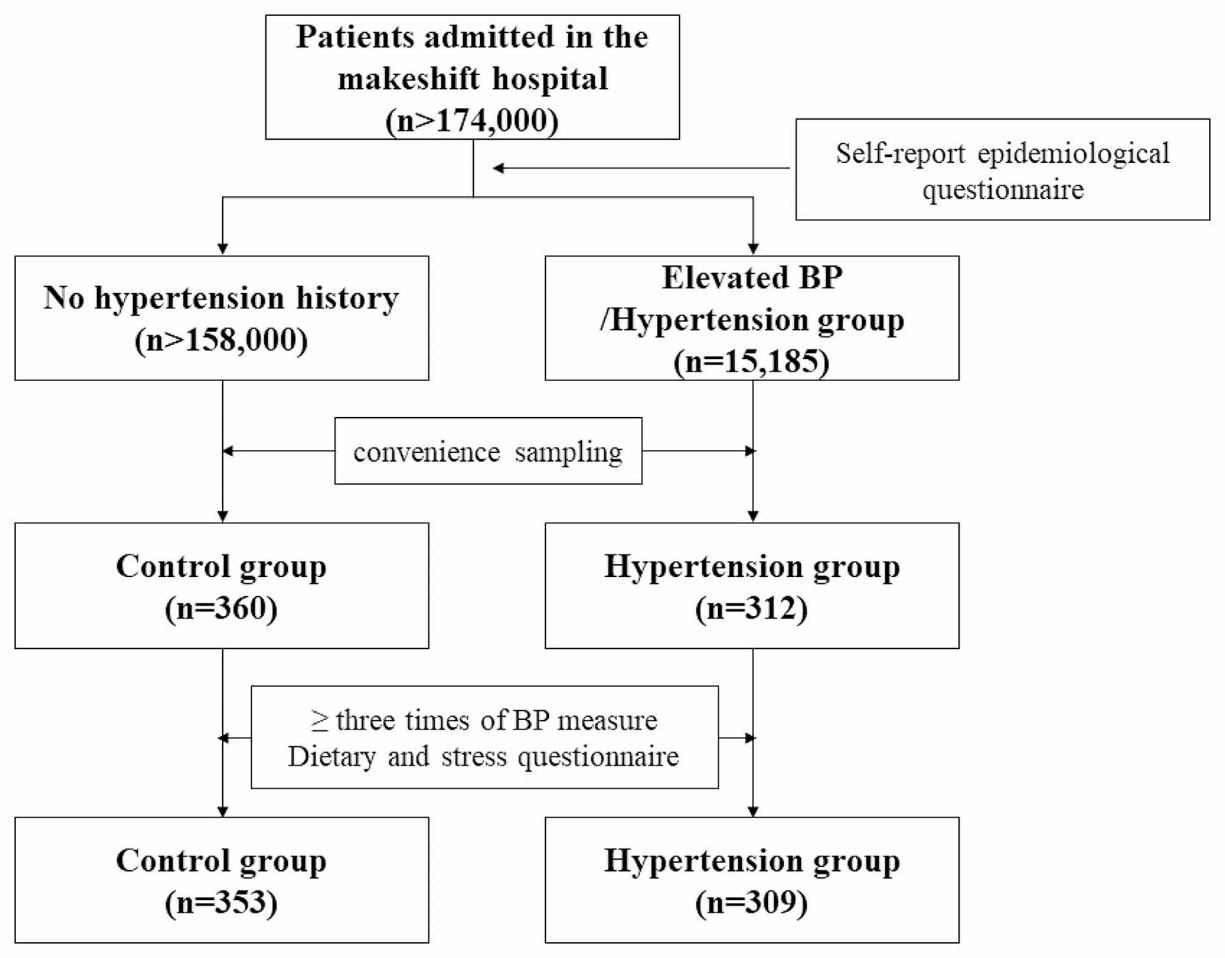
Flow chart of participant enrollment and follow-up

This was a cross-sectional study conducted in a centralized makeshift hospital that had been converted from the National Exhibition and Convention Center in Shanghai. The hospital started to receive Omicron-infected patients on April 9, 2022. As of May 31, when it closed, more than 174,000 patients had been admitted and received treatment there during the outbreak. All patients were asked to fill out a self-report epidemiological questionnaire via the WeChat-based survey program Questionnaire Star. Completing the survey took between 5 and 10 min. One of the items was about past medical history, which was used to gather information on underlying diseases such as hypertension, diabetes, and coronary heart disease. A total of 15,185 patients claimed that they had elevated BP or hypertension. Participants of the study were recruited through convenience sampling. The researchers explained the purpose and major items of the study to the patients by face-to-face communication or telephone consultation, and obtained their informed consent. The patients were then supplied with an electronic questionnaire on diet and stress by scanning a Quick Response code. Some elderly patients and patients without smartphones received instructions about the survey and help to complete it by the investigators at the bedside, or their data were filled via telephone contact. The recruited patients were told to visit the nurse’s station each morning to have their BP taken. Patients were required to complete BP monitoring at least three times. Their daily BP values were recorded and submitted online by scanning a Quick Response code, or by telephone contact from the investigators. Finally, a total of 662 patients successfully finished the study procedures with informed consent obtained, all of whom were included in the data analysis (Fig. 1). The study was conducted according to the principles of the Declaration of Helsinki. The procedures were approved by the Institutional Ethics Committee of Daping Hospital (2022 97 − 01), Army Medical University.

This study examined patients aged 18 years and older who were diagnosed with COVID-19 by real‐time fluorescence quantitative polymerase chain reaction assay using pharyngeal/nasopharyngeal swab specimens. Only asymptomatic patients and a small proportion of patients with mild flu-like symptoms were included. Patients were asked if there was any source of psychological stress that would have caused a change in their psychosocial status other than COVID-19 in the previous year. Patients with at least one of the following were excluded: type 1 diabetes mellitus, hypertensive emergencies, history of tumors; pregnant or lactating women; combination of diseases affecting cognitive function (congenital dementia; traumatic brain injury; severe cardiac, renal, or pulmonary dysfunction; epilepsy; severe hypoglycemia; coma; cerebrovascular disease; or ischemic heart disease); alcohol and drug addiction, mental illness, or psychoactive drug abuse; visual, auditory, or language impairment; or an inability to cooperate with the completion of the dietary and stress questionnaires.

Office BP (patients’ BP measured during hospitalization) measurements were performed using a validated automated electronic sphygmomanometer (Omron HEM-7136). Patients were asked to rest for a few minutes in a seated position with uncrossed legs. The average of two consecutive measurements taken 1 to 2 min apart was defined as the office BP. Then, patients with hypertension were divided into controlled (reading < 140/90 mmHg) and uncontrolled (reading ≥ 140/90 mmHg) groups based on the office BP, according to the clinical guideline [23]. Normal BP controls were screened through patients’ self-reported past medical history, i.e., with no history of hypertension. Those patients were labeled as having raised BP if their readings were greater than 140/90 mmHg on different days for three or more times during hospitalization.

### Dietary and stress questionnaire

The diet questionnaire was adopted and expanded from our previous work [22, 24]. The original version included 19 items of general information and 8 items evaluating food preferences. The food preference questionnaire included items that elicited participants’ self-reported preferences for salty and spicy foods in their daily life. We had supplemented the general information content to allow for a more detailed assessment of the impact of the outbreak on the patient’s daily life (including sleep, diet, respiratory symptoms) and a detailed patient history of hypertension (anti-hypertensive drugs, course of hypertension, family history, and history of gestational hypertension for women).

The HAMA and HAMD questionnaires were used to evaluate the anxiety and depression levels of the patients [25]. Patients were defined as having mild to moderate anxiety or depressive symptoms if they scored ≥ 7 on the HAMA or HAMD, respectively. Higher scores indicate increased disease severity and vice versa.

### Habitual SP evaluation

For the assessment of habitual SP, we mainly used the dietary questionnaire-derived method of our previous study[22]. In brief, we used the 10 most common salty foods eaten in Shanghai—fried pork slices with salted pepper, sautéed sliced pork, spicy poached slices of pork or fish, hot pot, salted pickles, bean paste, pickled Chinese cabbage, bacon, barbecue, and salted eggs. The participants were instructed to report their frequency of eating these foods in daily, weekly, or monthly periods. For each type of food, the calculated frequency was expressed as follows: (1) daily: 30, 60, and 90 times; (2) weekly: 4, 8, 12, 16, 20, and 24 times; and (3) monthly: 0, 1, 2, and 3 times. Shanghai residents, in contrast to our earlier study, like sweet food and often consume less salty food. Thus, the grouping for SP was based on the frequency of the consumption of salty foods, i.e., low SP with a frequency of no more than 5 times; and high SP with a frequency of more than 5 times. For the participants who reported eating more than one type of salty food, the total frequency was further divided by the number of food types, which yielded the average frequency of salty food intake. We also took into account the potential impact of regional differences in diet, and subjective SP was included in the evaluation. Using two food preference items, we were able to determine self-reported SP. Finally, habitual SP (as high SP) was derived from frequency of the consumption of salty foods and self-reported SP.

### Outcome measurements

The primary outcome in this analysis was the synergistic effect of Omicron epidemic-related stress and habitual SP on BP profiles in COVID-19 patients with hypertension. Office BP measurements were considered as valid BP data if they had been taken at least three times per patient during hospitalization. The secondary outcome included the effects of Omicron epidemic-related stress and habitual SP on the average hospital stays of COVID-19 patients.

### Covariates

We collected the demographic, dietary, psychological stress, and medical history information using the online self-administered questionnaire and the electronic medical record of the makeshift hospital. We included the following covariates in the main analysis: age (years), sex (male or female), education (with or without college graduation), work status (light and heavy physical labor), body mass index (calculated as weight divided by height squared, kg/m^2^), cigarette smoking (never, former, or current), alcohol drinking (never/seldom, former, or current), sleep quality (as normal, worse), appetite (as normal, worse), flu-like symptoms (cough, sore throat, nasal congestion, fatigue: yes or no), comorbidities (diabetes mellitus, dyslipidemia, cardiovascular disease: yes or no), days from COVID-19 diagnosis to hospital admission, course of hypertension, use of antihypertensive drugs (ARB/ACEI, Ca^2+^ channel blocker, β blocker, thiazide, mineralocorticoid receptor blocker, loop diuretic), and family history of hypertension.

### Statistical analysis

Results are presented as mean ± standard deviation (SD), n (%), or mean and 95% confidence interval (CI). The baseline characteristics of the participants were compared between the groups using the χ^2^ test for categorical variables and the independent two-sample t-test for continuous variables. Multivariable adjustment for age, sex, educational level, body mass index, sleep, eating, HAMA and HAMD scores, as well as the corresponding 95% CIs, were estimated by covariance analysis using a univariate general linear model. Model 1 was adjusted for age, sex, educational level, and body mass index. Model 2 was adjusted for the Model 1 covariates and for sleep and eating. Model 3 was adjusted for the Model 2 covariates and for HAMA and HAMD scores. Numerical statistical analyses were conducted using SPSS software, version 13.0 (SPSS, Inc.) or GraphPad Prism software, version 5.0 (GraphPad Software), and a two-sided P value of < 0.05 was considered statistically significant.

## Results

### Hypertensive patients did not show anticipated epidemic-related social stress

In this study, a total of 662 patients with asymptomatic and mild Omicron infection were included in the main analysis. Table 1 shows the baseline characteristics of the enrolled patients, with 353 patients self-reporting no history of hypertension (control group). The duration of hypertension history of the 309 hypertensive patients was 7.4 ± 7.0 years, with mean (SD) BP 134 (11)/85 (7) mmHg. Compared with the controls, patients with hypertension tended to be older, male, less educated, of higher BMI, and more stressed at work. Only 2 (0.6%) of the controls and 3 (1%) from the hypertension group had HAMA scores ≥ 7, and 5 (1.6%) hypertensive patients had HAMD scores ≥ 7. Thus, the Omicron epidemic did not have the anticipated large impact on social stress. Of significance, a higher proportion of hypertensive patients had habitual SP (*P* = 0.015) and higher HAMA (*P* < 0.001) and HAMD (*P* < 0.001) scores. In contrast, patients without a history of hypertension were more prone to experience sleep (*P* < 0.001) and eating (*P* < 0.001) disorders being impacted by COVID-19. As expected, hypertensive patients had longer hospital stays (7.4 ± 1.5 days vs. 7.2 ± 0.5 days, *P* = 0.019).

**Table 1:** Baseline characteristics of the enrolled patients with asymptomatic and mild omicron infection

	Control (*n* = 353)	Hypertension (*n* = 309)	***P*** value
Age, y	40.1 ± 13.5	56.6 ± 9.7	< 0.001
Male, n (%)	241 (68.3)	253 (81.9)	< 0.001
College degree and above, n (%)	123 (34.8)	49 (15.9)	< 0.001
Body mass index, Kg/m^2^	23.1 ± 3.4	25.6 ± 3.3	< 0.001
Duration of hypertension, y	NA	7.4 ± 7.0	NA
Systolic BP, mmHg	116 ± 12	134 ± 11	< 0.001
Diastolic BP, mmHg	77 ± 9	85 ± 7	< 0.001
Work stress, n (%)			< 0.001
Less	268 (75.9)	299 (96.8)	
Moderate	59 (16.7)	6 (1.9)	
High	26 (7.4)	4 (1.3)	
Salty preference, n (%)			0.015
Low	199 (56.4)	145 (46.9)	
High	154 (43.6)	164 (53.1)	
HAMA score	0.2 ± 1.0	0.8 ± 1.6	< 0.001
HAMD score	0.2 ± 0.7	0.8 ± 1.5	< 0.001
Impaired sleep, n (%)	83 (23.5)	20 (6.5)	< 0.001
Impaired eating, n (%)	52 (14.7)	8 (2.6)	< 0.001
Hospitalization time, d	7.2 ± 0.5	7.4 ± 1.5	0.019

Differences between groups were compared using the χ^2^ test for categorical variables and the independent two-sample t-test for continuous variables, and a two-sided *P* value of < 0.05 was considered statistically significant

### Habitual SP contributed to worsen BP control both in omicron infected patients with and without hypertension, and psychosocial stress were associated with elevated BP during hospitalization

Table 2 shows the association of habitual SP and BP with Omicron epidemic-related stress in COVID-19 patients. We found that habitual SP was associated with higher BP both in patients with and without hypertension, after adjusting for a wide range of covariates, including Omicron epidemic-related stress (HAMA and HAMD scores). Hypertensive patients with habitual SP were found to have an increase of both systolic (4.9 [95%CI, 2.3–7.4], *P* < 0.001) and diastolic (2.1 [95%CI, 0.6–3.6], *P* = 0.006) BP during the Omicron epidemic, compared with those patients who reported non-habitual SP. Patients with no previous history of hypertension showed different outcomes. Habitual SP was found to be more significantly associated with increased diastolic BP (2.0 [95%CI, 0.2–3.7], *P* = 0.026) whereas only a tendency of increased systolic BP (2.2 [95%CI, (0.1–4.6], *P* = 0.064) was observed after adjusting for HAMA and HAMD scores.

**Table 2:** Association of habitual SP and BP in patients

	Control (*n* = 353)		Hypertension (*n* = 309)	
	non-habitual SP	Habitual SP	non-habitual SP	Habitual SP
Unadjusted				
Systolic BP, mmHg	Ref	2.9 (0.3 to 5.6) (*P* = 0.027)	Ref	4.1 (1.6 to 6.7) (*P* = 0.001)
Diastolic BP, mmHg	Ref	2.7 (0.7 to 4.3) (*P* = 0.008)	Ref	1.9 (0.4 to 3.4) (*P* = 0.014)
Model 1				
Systolic BP, mmHg	Ref	2.6 (0 to 5.2) (*P* = 0.047)	Ref	5.0 (2.4 to 7.5) (*P* < 0.001)
Diastolic BP, mmHg	Ref	2.3 (0.5 to 4.1) (*P* = 0.011)	Ref	2.2 (0.7 to 3.7) (*P* = 0.004)
Model 2				
Systolic BP, mmHg	Ref	2.8 (0.3 to 5.3) (*P* = 0.03)	Ref	4.9 (2.4 to 7.4) (*P* < 0.001)
Diastolic BP, mmHg	Ref	2.4 (0.6 to 4.1) (*P* = 0.010)	Ref	2.2 (0.6 to 3.7) (*P* = 0.005)
Model 3				
Systolic BP, mmHg	Ref	2.2 (0.1 to 4.6) (*P* = 0.064)	Ref	4.9 (2.3 to 7.4) (*P* < 0.001)
Diastolic BP, mmHg	Ref	2.0 (0.2 to 3.7) (*P* = 0.026)	Ref	2.1 (0.6 to 3.6) (*P* = 0.006)

Multivariable adjustment for age, sex, educational level, body mass index, sleep, eating, HAMA and HAMD scores, as well as the corresponding 95% CIs, were estimated by covariance analysis using a univariate general linear model. Model 1 includes adjustment for age, sex, educational level, and body mass index. Model 2 adjusts for model 1 parameters, as well as sleep and eating. Model 3 adjusts for model 2 parameters and HAMA: HAMD scores. CI: confidence interval

During hospitalization, 31 (8.8%) patients with no prior history of hypertension developed increased BP. We contrasted those patients with the other patients in the control group (Table 3). We found that the patients who developed elevated BP during hospitalization were of relatively older age, had higher BMI and work stress, and were more prone to sleep disorders. Higher HAMA and HAMD scores were found in the patients with elevated BP during hospitalization, whereas no differences in habitual SP were observed in them compared with patients with normal BP (*P* = 0.576). In contrast, habitual SP was more common in hypertensive patients with uncontrolled BP, compared with patients with controlled BP (Table 3, *P* = 0.002). No differences were seen in HAMA and HAMD scores, sleep and eating disorders, and work stress in hypertensive patients with or without BP controlled (Table 3). Furthermore, uncontrolled BP was relatively common in male (*P* = 0.001) hypertensive patients with higher BMI (*P* = 0.022).

**Table 3:** Contribution of habitual SP and stress to the BP control in patients

	Control (*n* = 353)			Hypertension (*n* = 309)		
	Normal BP (*n* = 322)	Raised BP (*n* = 31)	***P*** value	**Controlled** (*n* = 176)	Uncontrolled (*n* = 133)	***P*** value
Age, y	39.7 ± 13.6	45.2 ± 11.9	0.028	55.9 ± 9.8	57.4 ± 9.4	0.182
Male, n (%)	217 (67.4)	24 (77.4)	0.252	133 (75.6)	120 (90.2)	0.001
College degree and above, n (%)	111 (34.5)	12 (38.7)	0.636	31 (17.6)	18 (13.5)	0.331
Body mass index, Kg/m^2^	22.9 ± 3.3	24.8 ± 3.2	0.002	25.2 ± 2.9	26.1 ± 3.7	0.022
Systolic BP, mmHg	114 ± 10	140 ± 14	< 0.001	127 ± 8	144 ± 8	< 0.001
Diastolic BP, mmHg	76 ± 7	94 ± 8	< 0.001	81 ± 5	90 ± 6	< 0.001
Duration of hypertension, y	NA	NA	NA	7.4 ± 6.8	7.6 ± 7.3	0.803
Work stress, n (%)			0.036			0.722
Less	250 (77.6)	18 (58.1)		170 (96.6)	129 (97.0)	
Moderate	51 (15.8)	8 (25.8)		3 (1.7)	3 (2.3)	
High	21 (6.5)	5 (16.1)		3 (1.7)	1 (0.8)	
Salty preference, n (%)			0.576			0.002
Low	183 (56.8)	16 (51.6)		96 (54.5)	49 (36.8)	
High	139 (43.2)	15 (48.4)		80 (45.5)	84 (63.2)	
HAMA score	0.1 ± 0.8	1.5 ± 1.9	< 0.001	0.3 ± 0.4	0.2 ± 0.4	0.525
HAMD score	0.1 ± 0.4	1.4 ± 1.7	< 0.001	0.3 ± 0.5	0.3 ± 0.5	0.751
Impaired sleep, n (%)	69 (21.4)	14 (45.2)	0.003	11 (6.3)	9 (6.8)	0.855
Impaired eating, n (%)	44 (13.7)	8 (25.8)	0.068	3 (1.7)	5 (3.8)	0.297

Differences between groups were compared using the χ^2^ test for categorical variables and the independent two-sample t-test for continuous variables, and a two-sided *P* value of < 0.05 was considered statistically significant

## Discussion

To the best of our knowledge, this is the first and largest study evaluating the synergistic effect of stress and habitual SP on BP in the hospitalized Omicron-infected patients during the COVID-19 pandemic. Our results indicate that habitual SP and higher HAMA and HAMD scores were associated with higher BP in Omicron-infected patients with or without hypertension. We found that the effect of habitual SP on BP remained stable in hypertensive patients, and on diastolic BP in controls after adjusting for covariates including Omicron epidemic-related stress. Furthermore, habitual SP contributed to uncontrolled BP in hypertensive patients, whereas Omicron epidemic-related stress was associated with elevated BP in controls.

### Potential mechanism

The COVID-19 pandemic exerted a negative influence on human mental health, and an increase in daily stress was expected to occur, especially in chronically ill patients who were already experiencing stress because of their illness. When humans are exposed to stress, two main physiological systems are activated [26, 27]. Activation of the sympathetic nervous system coupled with the adrenal medullary system and activation of the hypothalamo-pituitary adrenal axis all contribute to the increase of BP through the release of stress-related hormones such as cortisol, catecholamines, and norepinephrine. Furthermore, dysregulation in the Renin–Angiotensin System (RAS) was linked to the systemic manifestations of COVID-19, which has key roles in the regulation of vascular tone, electrolyte balance, inflammation, thrombosis and response to injury [28, 29].

Strong evidence exists for a causal relationship between salt intake and BP, with underlying mechanisms related to fluid homeostasis, hormonal and inflammatory mechanisms, immune response, and the gut microbiome [30, 31]. High salt intake could lead to vascular dysfunction, defined by decreased NO production, cellular stiffening, and pressure-independent vascular remodeling [32]. Endothelial dysfunction was also suggested as an important mechanism for developing a series of systemic manifestations by COVID-19. SARS-CoV-2 can bind to angiotensin-converting enzyme 2 (ACE2) and being internalized in the endothelial cells, which in turn increasing the production of ROS and inflammatory cytokines (like IL-1, IL-6, and TNF), decreasing the bioavailability of NO and PGI2, and inducing endothelial cell apoptosis, and thereby results in endothelial damage and dysfunction [33–35]. Therefore, a high-salt diet with COVID-19 may increase the risk of vascular damage and cause blood pressure to fluctuate [36].

Experimental studies have suggested that early high salt exposure may increase stress sensitivity, involving changes to the structural plasticity of the prefrontal cortex (PFC) and nucleus accumbens (NAc) [13]. Both the PFC and NAc have been found to be involved in reward processing [37], and our previous work found that those areas also participated in high SP [22, 24]. Another study found that neuronal and behavioral responsivity to psychogenic stressors was potentiated by prior exposure to high salt intake [38]. This synergistic effect was suggested to be associated with activation of neurons producing vasopressin in the hypothalamic paraventricular nucleus and V1 receptors in the amygdala. Furthermore, a study found that high dietary salt intake could augment behavioral hyper-responsivity to psychological stress by promoting neuroinflammation and increasing recruitment of neurons in the same brain areas [14].

### Comparison with other studies

Tamura and colleagues published two works about the impact of the state of emergency owing to COVID-19 on stress and BP in Japan in 2022 [39, 40]. They found that office BP significantly increased during the state of emergency, accompanied by a higher ratio of white coat hypertension. However, the subjects in their studies were mostly diabetic patients, and only slight increases in BP of 1–2 mmHg were observed. The prevalence of many stress-related conditions, including anxiety and depression, was reported to be 17–20% higher in people with diabetes mellitus [41–43]. Therefore, it is difficult to distinguish whether psychological stress was due to the COVID-19 epidemic or its own existence contributed to BP increase. In contrast, another study also performed in a Japanese population with diabetes found that BP did not rise during the state of emergency but did rise significantly afterwards [11]. In this study an apparent relationship between BP increases and changes in diet or physical activity was not observed. In consideration of the previous findings that BP is raised significantly after major stresses such as an earthquake [44], the authors also speculated that psychological stress caused by the COVID-19 pandemic and prolonged restrictions to daily life could affect BP values. In this study, we used HAMA and HAMD scores to evaluate psychological stress induced by the COVID-19 epidemic in patients with no history of hypertension. Interestingly, we did not find a high prevalence of anxiety or depression among patients in the makeshift hospital. However, even in those individuals who merely had an emotional influence from the COVID-19 outbreak, we still discovered a significant rise in BP. Those patients had slightly higher HAMA and HAMD scores, and were prone to eating and sleep disorders. Importantly, the raised BP was mainly caused by epidemic-related stress, not by habitual SP, in previous healthy patients.

Three studies conducted thus far have concerned the effect of the COVID-19 pandemic on BP in hypertensive patients. Celik et al. found that psychological stress owing to the COVID-19 outbreak led to worsening of the regulation of 24-hour BP in controlled hypertensive patients whose antihypertensive treatments did not change [45]. We also found that patients with hypertension had relatively higher HAMA and HAMD scores, thus were prone to psychological stress during the COVID-19 pandemic. In contrast to our study, patients included in Celik’s study had no history of COVID-19 infection or vaccine. Pengo et al. assessed the effects of lockdown during COVID-19 on home BP control of hypertensive patients [46]. They found that home BP decreased after the COVID-19 lockdown, and this was most evident in those patients with uncontrolled high BP before lockdown. This was consistent with findings in diabetic patients that home BP decreased significantly during the state of emergency in Japan [39, 40]. A nationwide home BP monitoring study performed in Brazil also found no major adverse influence of the COVID-19 pandemic on office and home BP control [47]. The authors suggested that physical and psychological relaxation associated with lockdown may have prevailed over COVID-19-related stress in hypertensive patients. Consistent with those findings, we also found that hypertensive patients with uncontrolled BP did not experience heavy psychological stress compared with those hypertensive patients with controlled BP.

Consumption of high-sodium food has been used as a means of relieving mental stress during the pandemic. Dietary changes owing to the lockdown during the pandemic had been noted, with increased population preferences for processed and preserved food, which have a high sodium content that is not inherent to raw food ingredients but is added during their processing procedures [48]. A web-based survey conducted during the early COVID-19 containment phase in France reported an increase of up to 28.4% in caloric/salty food intake [17]. Studies on university students from seven South East Asian countries had revealed that during the pandemic, over 50% of students had a high salt intake [49, 50]. A study conducted in India involving children aged 9–14 years found that 25% of them consumed ultra-processed foods with high levels of salt during the pandemic [51]. Currently, we lack direct evidence of the effects of changes in salt intake on BP owing to COVID-19 pandemic-related stress. Although they reported a significant increase in BP in diabetic patients owing to COVID-19-related stress, Tamura et al. found no change in estimated salt intake before and after the state of emergency in Japan [39, 40]. In our study, habitual SP contributed to the rise in BP during the COVID-19 pandemic in Omicron-infected patients with and without hypertension. In hypertensive patients with habitual SP, systolic BP increased 4.9 mmHg and diastolic BP increased 2.1 mmHg during the Omicron epidemic, after adjusting for covariates including stress. Furthermore, habitual SP was associated with uncontrolled BP in hypertensive patients. However, in patients with no history of hypertension, the increase of BP during the COVID-19 pandemic was not related to habitual SP.

### Strengths of this study

This study has several important strengths. First, we targeted Omicron patients who were either asymptomatic or who had mild flu-like symptoms, to explore the synergistic effect of stress and habitual SP on BP. Our findings will be valuable in developing prevention and treatment measures for BP management of patients with COVID-19 infection in the future. Second, patients in this study were all in the same residential and therapeutic environment, which could effectively avoid the influence of confounding factors (temperature, food, exercise, etc.) and made it easier to observe the impact of stress and habitual SP on BP. Furthermore, we explored the use of online tools to study the impact of habitual SP and stress on BP during emergency events in a large patient’s size, which is timelier and more convenient than other methods. Last but not least, this study suggests that in order to achieve BP goals when managing hypertensive patients in sudden emergency events like COVID-19, it is necessary to take into account the synergistic effect of the event itself and the patient’s own BP influencing factors.

### Limitations of this study

This study has several limitations. First, the limitation to the interpretation of these findings is the cross-sectional nature of the study, and only patients who admitted in the hospital were included. The city was under lockdown during the course of this study, therefore a control group of hypertensive individuals who were not infected with COVID-19 was not recruited for comparison. Thus, the representativeness and external validity of the findings need to be further confirmed. Second, the self-administered questionnaire could only provide qualitative measurement of SP, with detailed dietary information unavailable. Conducting a salt taste test and daily salt intake monitoring, as in our previous work [22] would be more accurate. However, owing to the high daily turnover of patients in the makeshift hospital during the pandemic, much more manpower and time for follow-up would have been required. Third, other risk factors for BP increase that may have changed during the pandemic, such as daily exercise and food types, were not analyzed in the present study. Fourth, we used convenience sampling to enroll patients due to the large number of hospitalized patients, which may lead to biased results. Fifth, missing items in patients’ feedback were not analyzed, such as cigarette smoking and alcohol drinking, along with the other factors like relationships could also impact BP outcomes. Sixth, patients with increased BP during hospitalization who self-reported no history of hypertension, cannot be further identified as a cause of COVID-19, white-coat hypertension, etc. Finally, we did not investigate the long-term synergistic effects of the COVID-19 outbreak and habitual high salt intake on BP control, especially investigation of the return of BP to normal levels.

## Conclusions

We found that habitual SP and psychosocial stress were associated with higher BP in Omicron-infected patients with or without hypertension. The effect of habitual SP on BP remained stable in hypertensive patients, and on diastolic BP in controls under various levels of psychosocial stress. Furthermore, our findings suggested that habitual SP contributed to uncontrolled BP in hypertensive patients, and Omicron epidemic-related stress was associated with raised BP in controls. This highlights the important role of NPIs including dietary guidance and psychiatric therapy are crucial for BP control during the long COVID-19 period. Based on our previous research findings, dietary intervention strategy such as adding spices can be used to reduce salt intake [22]. These strategies have been recommended in several guidelines as a means of preventing cardiovascular diseases in general population [52–54].

## Data Availability

The data generated during the current study are available from the corresponding author on reasonable request.
